# Comprehensive Evaluation of a Levonorgestrel Intrauterine Device (LNG-IUD), Metformin, and Liraglutide for Fertility Preservation in Endometrial Cancer: Protocol for a Randomized Clinical Trial

**DOI:** 10.3390/life14070835

**Published:** 2024-06-29

**Authors:** Gergő Leipold, Richárd Tóth, Péter Hársfalvi, Lotti Lőczi, Marianna Török, Attila Keszthelyi, Nándor Ács, Balázs Lintner, Szabolcs Várbíró, Márton Keszthelyi

**Affiliations:** 1 Department of Obstetrics and Gynecology, Semmelweis University, 1082 Budapest, Hungary; leipold.gergo@semmelweis.hu (G.L.); toth.richard@semmelweis.hu (R.T.); keszthelyi.lotti.lucia@semmelweis.hu (L.L.); torok.marianna@semmelweis.hu (M.T.); acs.nandor@semmelweis.hu (N.Á.); lintner.balazs.zoltan@semmelweis.hu (B.L.); varbiro.szabolcs@semmelweis.hu (S.V.); 2 BiTrial Clinical Research, 1121 Budapest, Hungary; peter.harsfalvi@bitrial.hu; 3 Workgroup of Research Management, Doctoral School, Semmelweis University, 1085 Budapest, Hungary; 4 Department of Urology, Semmelweis University, 1082 Budapest, Hungary; keszthelyi.attila@semmelweis.hu; 5 Department of Obstetrics and Gynecology, University of Szeged, 6725 Szeged, Hungary

**Keywords:** endometrial cancer, fertility preservation, LNG-IUD, metformin, liraglutide, gynecology oncology

## Abstract

Endometrial cancer is a leading gynecological malignancy, with obesity being a significant risk factor due to increased estrogen production in body fat. Current treatments often involve hysterectomy, which precludes fertility, thus highlighting the need for fertility-preserving options. This study aims to evaluate the combined efficacy of a levonorgestrel intrauterine device (LNG-IUD), metformin, and liraglutide for treating women with endometrial hyperplasia or early stage endometrial cancer while preserving fertility. The study will enroll 264 women aged 18–45 with a BMI > 30 who desire uterine preservation. Participants will be randomized into three groups: LNG-IUD alone, LNG-IUD plus metformin, and LNG-IUD plus metformin and liraglutide. Primary outcomes will include complete pathological remission, while secondary outcomes will assess histological changes, glucose, insulin levels, and weight changes over a 12-month period. This study protocol hypothesizes that LNG-IUD combined with metformin and liraglutide may potentially lead to higher regression rates of endometrial hyperplasia (EH) and early stage endometrial cancer (EC) compared to LNG-IUD alone. Furthermore, the protocol anticipates that these combination therapies will demonstrate good tolerability with minimal adverse effects, suggesting the potential benefit of integrating metabolic interventions with LNG-IUD to enhance treatment efficacy while preserving fertility in women with EH and EC.

## 1. Introduction

Endometrial cancer (EC) stands as a prevalent concern in developed societies, ranking as the most common gynecological cancer globally and the sixth most common overall [1,2]. Estimations project a potential 50% surge in global cases by 2040, highlighting the urgency to redefine its management [3]. The estrogen dependence of 70–80% of EC types links to the presence of aromatase in body fat, fostering heightened estrogen production with increased body fat levels. This notably elevates the risk of EC, with women categorized as obese or severely obese facing 2.6 to 4.7 times higher likelihoods of developing the disease compared to those with normal weight [4,5].

Even in the early stages, conventional therapy involves the removal of the uterus, fallopian tubes, and sentinel lymph nodes; and, in some cases, systematic lymph node dissection. However, this approach excludes future fertility prospects impacting around 14% of premenopausal women, including 5% younger than 40 years old [5]. Therefore, the demand for fertility-preserving therapies is markedly amplified [6], although these methods mean a diversion from standard guidelines, which needs a holistic, multidisciplinary approach in every single patient’s case [7,8]. When deciding about fertility sparing, it is crucial that the desire for childbearing and reproductive capability are still present despite the diagnosis.

To address this critical need, the concept of using progesterone preparations, particularly intrauterine devices (IUDs) containing LNG-levonorgestrel, gained traction. These devices ensure continuous progesterone release within the uterine cavity, offering a more favorable side effect profile compared to oral progesterone formulations [9]. Simultaneously, recognizing the imperative to mitigate excess estrogen dominance, aggressive weight-loss therapy emerges as an essential adjunct to reduce tumorous growth. 

Metformin and Glucagon-like peptide-1 (GLP-1) agonists, which have been attracting increasing attention lately, offer promising approaches for reducing the risk of endometrial cancer by impacting molecular and metabolic pathways. Metformin, by activating AMP-activated protein kinase (AMPK) and inhibiting the mTOR pathway, can suppress cell proliferation and induce apoptosis in endometrial cancer cells [10,11]. Additionally, metformin’s ability to improve insulin sensitivity and reduce insulin levels addresses metabolic dysregulation, a key driver of endometrial cancer [12]. 

GLP-1 agonists exhibit antitumor effects by inhibiting inflammatory processes, which are significant because chronic inflammation is linked to the development and progression of various cancers. Additionally, GLP-1 agonists activate intracellular cAMP pathways, leading to cell cycle arrest and the inhibition of cell proliferation and tissue invasion, thereby counteracting cancer cell growth and metastasis [13,14]. Furthermore, GLP-1 agonists’ impact on weight loss and metabolic parameters offers an additional layer of protection against endometrial cancer development in high-risk populations. Combined, these medications could present a multifaceted approach to reduce the risk of endometrial cancer by targeting both molecular and metabolic pathways involved in its pathogenesis [15].

While studies have explored the role of LNG-IUDs in managing early stage endometrial cancer and hyperplasia, there is a noticeable gap in research concerning the impact of metabolic intervention [16]. This underscores the necessity for additional investigations to understand how the combination of weight loss and LNG-IUDs affects the complete pathological response rate, emphasizing the need for further exploration of fertility preservation and tumor regression. This will be the first investigation to examine the role of liraglutide in such a patient group. 

Our overarching goal is to investigate the combined impact of progesterone preparations, metformin, and liraglutide-based weight loss therapy on patients with hyperplasia or early stage endometrial cancer. This approach aims to not only counterbalance estrogen dominance but also preserve fertility in affected individuals, paving the way for a novel, comprehensive treatment strategy in this population. One of our specific aims is to determine the combined effect of LNG-IUD, metformin, and liraglutide, and to assess the differences in their effectiveness. We aim to investigate whether metformin alone, or in combination with liraglutide, is more effective alongside the standard LNG-IUD treatment in achieving complete pathological remission.

Another key aim is to investigate the metabolic impact of these treatments and their relation to complete pathological remission. We will monitor glucose and insulin levels, as well as track weight loss (measured in kilograms) at set intervals during the 12-month check-up. This comprehensive assessment seeks to deeply understand how interventions interact with metabolic reactions, offering valuable insights into their influence on both physiological indicators and tangible results.

## 2. Experimental Design

This protocol was developed following the guidelines outlined in the Standard Protocol Recommendations for Interventional Trials (SPIRIT) reporting template [17]. This multi-center randomized 1:1:1 open-label clinical trial has three treatment groups: LNG-IUD, LNG-IUD plus metformin and LNG-IUD plus metformin and liraglutide therapy. The study protocol was approved and registered by the Ethics Committee of Semmelweis University (SE RKEB 63/2024). 

### 2.1. Study Setting

The study will involve women aged 18–45 diagnosed with endometrial hyperplasia or early stage endometrial cancer who meet the following criteria: (1) desire to retain their uterus and (2) obesity (BMI > 30). Early stage endometrial cancer criteria include stage 1, grade 1 disease with no lymphovascular space invasion and no myometrial invasion, as diagnosed by ultrasound or MRI. We plan to enroll 264 patients between January 2025 and December 2025.

### 2.2. Eligibility Criteria

#### 2.2.1. Inclusion Criteria

- Women in their reproductive years (18–45);
- sup>∙ Females with a body mass index (BMI) > 30 kg/m^2^;
- Histologically confirmed EHA or low-grade endometrioid endometrial cancer;
- No sign of myometrial invasion (confirmed by a gynecologist oncologist ultrasound or MRI—FIGO IA);
- No hypersensitivity or contraindications to LNG-IUD, MA, or metformin, or liraglutide;
- No use of metformin before inclusion at least for 2 years;
- Understanding the study design, risks and benefits, providing informed consent, and the ability to comply with the study protocol;
- Negative pregnancy test 7 days before starting the treatment.

#### 2.2.2. Exclusion Criteria

- Previous treatment for endometrial cancer;
- Advanced endometrial cancer (FIGO IA<);
- Non-endometrioid, or high-grade histology;
- Known allergies or intolerances to LNG-IUD, or diabetic medications;
- Inability to comply (exercise and attend on regular visits);
- Disorders other than diabetic endocrine disorders (renal disorder, liver failure);
- Lactation;
- Previous thrombosis, stroke, or acute myocardial infarction in the anamnesis.

Clinical characterization includes not only demographic information and clinical observation but also laboratory and histology testing. Enrolled patients undergo hysteroscopy to sample the entire endometrium. Women who had previously undergone dilatation and curettage (D&C) for this diagnosis were not required to undergo a second sampling, but the previous pathology specimen should be obtained by an expert gynecologic pathologist. 

### 2.3. Objectives

The primary objective of this study is to assess the efficacy of combined progesterone containing IUD and combines metformin and liraglutide therapy to achieve the complete pathology response rate histological results and define the role of metformin and liraglutide in the pathological response. To provide a thorough analysis, we will monitor glucose and insulin levels, and track weight loss over a 12-month period. This comprehensive assessment is designed to explore how these interventions interact with metabolic processes, thereby offering valuable insights into their impact on both physiological markers and overall health outcomes.

#### 2.3.1. Primary Outcome

The primary outcome is the complete pathological remission of endometrial hyperplasia or early stage endometrial cancer in response to the therapy of LNG-IUD plus metformin and combined with liraglutide. 

#### 2.3.2. Secondary Outcome

The secondary outcomes include:

Assessing histological changes compared to baseline after 3 months;

Assessing histological changes compared to baseline after 6 months;

Assessing histological changes compared to baseline after 9 months;

Monitoring changes in glucose and insulin levels during the 12-month checkup;

Tracking weight changes during the 12-month checkup;

Comparing the effectiveness between study groups.

### 2.4. Study Assessments and Procedures 

The study assessments and procedures performed at baseline and at 3-, 6-, 9-, and 12-month visits are summarized in Table 1.

### 2.5. Sample Size

For both endometrium hyperplasia and endometrial cancer groups, a parallel, 3-group design will be used to assess whether there is any difference in the rates between the groups. The two-sided hypotheses will be evaluated with the chi-square test, with an overall Type I error rate (α) of 0.05. For the sample size calculations, the response rate for arm A (LNG-IUD) is expected to be 58.9%, while the response rates for both arms B and C (LNG-IUD plus metformin and LNG-IUD plus metformin plus liraglutide) are expected to be 86.7% based on the results of [18]. To reach at least 80% power with equal group size, the sample size needed is 33/group. Additionally, 24% of the participants are expected to be drop out [19], resulting in an increased target sample size of 44 participants/group (total sample size = 264).

### 2.6. Statistical Methods

Where applicable, baseline participant characteristics will be presented as frequencies and proportions (%) for categorical data and the mean, standard deviation, median and interquartile range (IQR) for continuous data. As the study interventions are considered low-risk interventions, no interim analysis is planned. The statistical tests for the primary outcome variable will be two-tailed, and a value of *p* < 0.05 will be considered to indicate statistical significance. All analyses will be performed using R version 4.4.1.

### 2.7. Provision of Study Materials

The LNG-IUDs will be supplied by Richter Gedeon PLC, while all diabetic medications will be manufactured by Novo Nordisk and provided through their generosity for this study.

### 2.8. Recruitment

Patients meeting the eligibility criteria will be recruited from gynecological oncology centers, coordinated by Semmelweis University, Hungary. Detailed information about the study will be provided verbally and in writing by the examining physician.

### 2.9. Implementation

The principal investigator or a designated sub-investigator will input the required data into the web-based allocation system for eligible patients. Subsequently, the system will allocate participants to either the LNG or LNG + metformin or LNG + metformin + liraglutide group. 

## 3. Procedures

### 3.1. Intervention

The levonorgestrel intrauterine device has been approved as a standard therapy in case of fertility-sparing procedures [20]. Compared to systematic progesterone therapy, adverse effects do not include nausea, thromboembolic complication, or weight gain [21].

A combination of 3000 mg (or maximum tolerated dose) of metformin will be administered to the study participants. To avoid gastrointestinal side effects such as diarrhea, bloating, and stomach pain, the metformin dose will be administered less rapidly, but reach the final dose within 2 months [22]. 

Liraglutide, a long-acting GLP-1 agonist, will be administered via subcutaneous injection once daily, irrespective of mealtimes or time of day. To enhance gastrointestinal tolerability, an initial dose of 0.6 mg will be used. After a minimum of one week, this dose should be escalated to 1.2 mg. For certain patients, further titration up to the maximum recommended dose of 1.8 mg daily may be beneficial [23].

Since all the patients in this trial will have a BMI over 30 kg/m^2^, medication of all three groups will include 2 × 2 g of Inositol, a diet plan comprising 160–200 g of carbohydrates, and a daily exercise routine of 40–60 min of walking. Exercise levels (sedentary, moderately active, and sufficiently active) will be measured using the Active Australia Survey [24].

### 3.2. Randomization 

**Figure 1:**
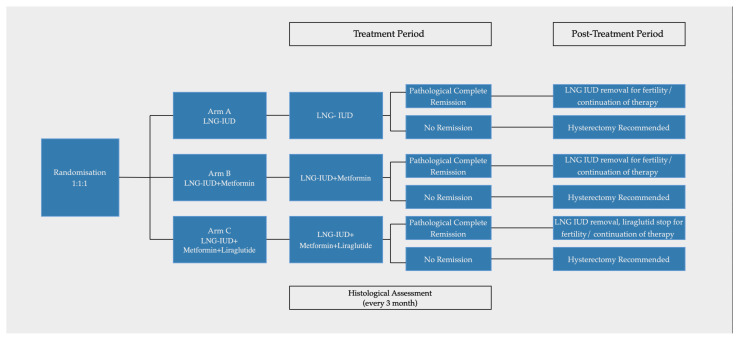
Trial design.

Patients will be randomly allocated in a 1:1:1 ratio to one of three groups: arm A (LNG-IUD), arm B (LNG-IUD plus metformin), or arm C (LNG-IUD plus metformin and liraglutide). This randomization will be conducted by an internet-based, remote third-party statistician who is blinded to the study and participant details. The recruiting physician will be trained and provided with detailed instructions on the recruitment protocol. The objective of randomization is to eliminate selection bias. Trial design is summarized in Figure 1. 

### 3.3. Treatment of Adverse Events

Local investigators will handle any adverse events in accordance with current good clinical practice guidelines. Each adverse event will be documented in a case report form, detailing its nature, onset and resolution times, severity, treatment, and outcome. Follow-up examinations may be conducted as needed to ensure patient safety. If a participant shows signs of harm or ineffectiveness, they will be removed from the study by the overseeing physician. These participants’ results will be analyzed separately as a non-pathological complete remission non-pCR group.

### 3.4. Criteria for Discontinuing or Modifying Allocated Interventions

The criteria for the discontinuation of trial medication are as follows.

1. A participant chooses to withdraw from the study or revokes their consent. Participants may leave the study at any time for any reason without facing consequences.
2. Cancelation of the entire study.
3. Discontinuation of the protocol treatment if it fails to achieve remission based on the following criteria: no treatment response or complete pathological response (pCR) within one year; disease progression at any time; or relapse after remission.
4. Occurrence of severe adverse events, potentially related to the medication (e.g., hemorrhagic shock due to massive vaginal bleeding, severe allergic reaction, thrombosis, liver function impairment), or the diagnosis of a new malignancy (e.g., breast cancer). These events will be assessed by two chief physicians before discontinuing the trial.
5. Any circumstance where the physician determines that treatment with LNG-IUD, metformin, or liraglutide cannot be continued.

### 3.5. Discontinuation of the Study

The study will be terminated early if the Institutional Review Board (IRB) identifies any of the following: serious adverse drug effects (e.g., unresponsive abnormal liver function, thrombosis, hemorrhagic shock due to massive bleeding, severe allergic reaction); diagnosis of a new malignancy; participants encountering unexpected, significant, or unacceptable risks (such as death); or the determination of treatment ineffectiveness.

### 3.6. Baseline Assessments 

Baseline assessments will adhere to the trial’s standard operating procedure (SOP). During the visit, a urine pregnancy test will be conducted. If the result is negative, LNG-IUD insertion will be performed following protocol guidelines. Blood tests and oral glucose tolerance tests will be administered at 0–60–120 min intervals to assess glucose and insulin levels. Education provided by a dietitian and physiotherapist will emphasize the importance of adopting healthy dietary habits and regular physical exercise. Participants will be encouraged to maintain an exercise diary. Medical consultation and the initiation of diabetic medications will also commence during this phase.

### 3.7. Efficacy Assessments 

Medical consultations regarding diabetic medication will occur in the second month to evaluate the tolerance of metformin or liraglutide. At 3, 6, 9, and 12 months following treatment initiation, a series of assessments will be conducted, including histology assessments to rule out disease progression and vaginal ultrasound assessments to measure endometrial thickness and invasion. If pathological complete remission(pCR) is attained, patients will receive guidance to remove the LNG-IUD and discontinue liraglutide if pregnancy is desired. Metformin may be continued without restrictions if tolerated.

### 3.8. Follow-Up Evaluations

During the 3-month follow-up, patients with progressive endometrial pathology will be withdrawn from the trial. Those showing complete response, partial response, or stable disease will continue to be monitored and undergo further evaluation after 3 months. At the 6-month follow-up, patients demonstrating progressive disease or recurrence in endometrial pathology will be withdrawn from the trial, while those with complete response, partial response, or stable disease will undergo another 3-month follow-up. Similarly, at the 9-month follow-up, patients exhibiting progressive disease or recurrence will be withdrawn, while those with complete response, partial response, or stable disease will be followed up again after 3 months. During the 12-month follow-up, patients with partial response, progressive disease, stable disease, or recurrence in endometrial pathology will be withdrawn from the trial and recommended for hysterectomy. Although the study duration is one year, treatment and patient follow-up will be long-term. Hysterectomy will be advised for patients who do not achieve complete pathological response within one year. Patients with fertility requirements or those unwilling to undergo hysterectomy will be closely monitored for signs of recurrence.

## 4. Data Monitoring Committee

An oversight committee comprising clinical trial experts, including a biostatistician, will be established. This committee will convene at least twice annually to review all trial data.

The oversight committee will analyze unblinded outcome data to assess safety and efficacy, determining whether there are indications of unsafe treatments warranting the discontinuation of the trial. Additionally, the committee will alert the Trial Steering Committee to any instances of unethical treatment or serious adverse events.

To manage the data for this study, the IBM Clinical Development Management System (IBM Corporation, Somers, NY, USA), utilizing an Electronic Data Capture System (EDC), will be employed.

## 5. Discussion

Our innovative study is the first to examine the combined effect of the levonorgestrel intrauterine device and combined weight reduction therapy, including metformin and liraglutide, on patients with a BMI > 30 who have low-grade and FIGO stage 1A endometrial cancer or endometrial hyperplasia and wish to preserve their uterus. This study aims to assess whether these new pharmaceutical interventions can increase remission rates compared to standard treatments, which focus on addressing the hormonal and metabolic imbalances associated with obesity and insulin resistance leading to EC. 

A study based on innovative avenues to diagnose and treat endometrial carcinoma [25] must consider various complexities, such as public health implications, ethical and legal aspects, and equitable access to care. Addressing these issues is crucial for the effective implementation of oncofertility counselling, which aims to support female cancer patients facing fertility threats due to cancer treatments [7]. Evidence-based oncofertility counselling requires a multidisciplinary approach in the form of an extended multidisciplinary tumor board involving medical oncologists, gynaecologists, pathologists, radiologists, reproductive endocrinologists, mental health counsellors, social workers, and clinical researchers to ensure comprehensive and informed decision making [25]. The integration of such services needs to adhere to international guidelines [8,26] to prevent medicolegal repercussions and ensure that ethical standards are upheld, enabling patients to make informed choices about their fertility preservation options [27]. This multidisciplinary framework underscores the moral obligation to support individual autonomy in medical decision making while ensuring that the welfare of potential offspring and others is safeguarded [28].

LNG-IUD has been established as the most effective form of progesterone for treating endometrial hyperplasia, with the narrowest profile of systemic side effects [29,30]. To complement the progesterone effect of LNG-IUD, metformin, an oral anti-diabetic drug, has been successfully used for its potential anti-cancer properties. Metformin activates adenosine monophosphate (AMP)-activated protein kinase (AMPK), affecting cellular metabolism and intracellular signaling by promoting the oxidative metabolism typical of quiescent cells and inhibiting the mTOR pathway [31,32]. This inhibition of mTOR, often activated in EC cells, helps overcome resistance to progestins and induces cell cycle arrest and apoptosis, suggesting metformin’s role as a promising therapeutic agent in EC treatment [33]. 

A meta-analysis comparing metformin plus LNG-IUD versus LNG-IUD monotherapy for treating endometrial hyperplasia suggests no clear difference in regression rates of hyperplasia between the two treatments, with very low-certainty evidence. Additionally, it remains unclear whether the combination therapy impacts rates of abnormal uterine bleeding, hysterectomy, or adverse effects. Therefore, the authors conclude that there is insufficient evidence to support or refute the use of metformin with standard therapy and emphasize the need for more robust and long-term randomized controlled trials to address this clinical question [34]. 

A retrospective analysis of 75 patients found that the fertility-sparing treatment of early stage endometrial cancer with LNG-IUD and metformin resulted in higher, though not statistically significant, complete response rates and effective fertility preservation. The authors suggest that the combination of megestrol acetate (MA), LNG-IUD, and metformin might be the most effective option for women desiring future pregnancies with low-risk early stage endometrial cancer [35]. 

Additionally, the glucagon-like peptide-1 (GLP-1) receptor agonist, liraglutide, has shown promising results not only in managing diabetes but also in cancer treatment. Although the literature on this topic is limited, liraglutide has been shown to be effective in reducing human prostate cancer incidence. Similarly, GLP-1 receptor agonists have been found to reduce in vitro proliferation and in vivo growth of prostate cancer cell lines [36]. A cohort study also indicated that people with Type 2 diabetes who took GLP-1Ras had a lower risk of developing colorectal cancer than those taking other medications [37]. However, initial studies have not yet extended to gynecological malignancies, particularly regarding fertility preservation.

## 6. Conclusions

Our study addresses an urgent need to combine anti-weight medication and the standard care of endometrial hyperplasia and endometrial cancer in fertility preservation to increase pathological remission. This clinical study is aimed to demonstrate that combining LNG-IUD with metformin and liraglutide significantly enhances the regression of endometrial hyperplasia and early stage endometrial cancer in patients with obesity while maintaining a favorable safety profile. The integrated approach of using metabolic therapy alongside localized hormonal treatment addresses the underlying risk factors associated with obesity and endometrial cancer, offering a promising fertility-preserving alternative to conventional surgical methods. 

## 7. Implication for Research 

The implications of this study for future research are profound. Firstly, the successful demonstration of the efficacy of the combined therapy could pave the way for larger, multi-center trials, potentially leading to a new standard of care for endometrial cancer patients. Secondly, the insights gained into the metabolic effects of metformin and liraglutide in conjunction with progesterone preparations can stimulate further investigation into their broader applications in oncology and metabolic diseases. Understanding the precise mechanisms through which these treatments affect pathological responses (e.g., interfering with miRNA-mRNA interactions [38]) will be crucial in optimizing therapeutic protocols and improving patient outcomes. Additionally, this research underscores the importance of integrating metabolic and oncological therapies, encouraging interdisciplinary collaboration and innovation. Future studies should also explore the long-term effects of these therapies on fertility and overall health, ensuring comprehensive care for patients. By prioritizing patient safety and meticulously monitoring treatment outcomes, this research sets a benchmark for clinical rigor and ethical responsibility in the development of new therapeutic strategies. Future clinical research should further explore long-term outcomes and potential benefits in broader patient populations, but these findings underscore the potential of combined therapeutic strategies in the management of endometrial cancer.

## Figures and Tables

**Table 1:** Schedule of patient assessments.

TIMELINE	Screening	Baseline	2*	3*	6*	9*	12*
Investigator meetings	X						
Informed consent and eligibility criteria	X						
Demographics and medical history	X						
Histology assessment by hysteroscopy/D&C/Pipelle	X						
MRI/Ultrasound of the pelvis	X						
Urine Pregnancy test		X					
LNG-IUD insertion		X					
Oral glucose tolerance test		X					X
Blood test		X					
Education by dietitian and physiotherapist		X					
Instructions for maintaining diet and exercise diary		X					
Medical consultation on diabetic medication		X	X				
Hysteroscopy sampling				X	X	X	X

## Data Availability

Data are contained within the article.
